# Adolescent Sexual Offenders: The Relationship Between Typology and Recidivism

**DOI:** 10.1177/1079063210369011

**Published:** 2010-06

**Authors:** Chi Meng Chu, Stuart D. M. Thomas

**Affiliations:** Monash University, Melbourne, Victoria, Australia, chimeng.chu@med.monash.edu.au, Clinical and Forensic Psychology Branch, Ministry of Community Development, Youth and Sports, Singapore; Monash University, Melbourne, Victoria, Australia, Victorian Institute of Forensic Mental Health, Melbourne, Victoria, Australia

**Keywords:** adolescent sexual abusers, juvenile sex offender, typology, delinquency, recidivism, victim

## Abstract

Adolescent sexual offending represents an ongoing social, judicial, clinical, and policy issue for services. The current study investigated the characteristics, criminal versatility, and rates of recidivism of a cohort of 156 male adolescent sexual offenders who were referred for psychological assessments by the courts between 1996 and 2007 in Singapore. Analyses revealed that specialists (sex-only offenders; * n* = 71, *M*_follow-up_ = 56.99 months, *SD*_follow-up_ = 31.33) and generalists (criminally versatile offenders; *n* = 77, *M*_follow-up_ = 67.83 months, *SD*_follow-up_ = 36.55) differed with respect to offense characteristics (e.g., sexually assaulting familial victims) and recidivistic outcomes. Although both groups sexually reoffended at roughly the same rate (14.3% vs. 9.9%), consistent with their typology, significantly more of the generalists reoffended violently (18.2% vs. 1.4%), sexually and/or violently (27.3% vs. 11.3%), nonviolently (37.7% vs. 16.9%), and engaged in any further criminal behaviors (45.5% vs. 23.9%) during follow-up. Adjusting for total number of offenses and age at first sexual offense, Cox regression analyses showed that generalists were significantly more likely than specialists to reoffend violently (hazard ratio = 9.31; 95% confidence interval = 1.15-76.39). The differences between generalists and specialists suggest a valid typological distinction with a higher risk trajectory for the generalists. These findings therefore have important clinical implications for assessment, management, and intervention planning for adolescent sexual offenders.

Sexual abuse is highly intrusive and is often associated with deleterious long-term psychological and physical outcomes for the victim (e.g., Boney-McCoy & Finkelhor, 1996; Campbell & Vasco, 2005; Noll, Horowitz, Bonanno, Trickett, & Putnam, 2003). Concerns about public safety have led to policies and clinical practices that are highly restrictive for the sexual offenders (Garfinkle 2003; Wood & Ogloff, 2006). The underlying assumption of these is that clinicians can accurately predict who will go on to reoffend, when this is not necessarily the case (Bengtson & Långström, 2007; Wood & Ogloff, 2006).

Studies on sexual offending among youths suggest that although the majority of adolescents who commit sexual offenses do not continue offending into their 20s, somewhere between 9% and 15% do (Nisbet, Wilson, & Smallbone, 2004; Rasmussen, 1999). Furthermore, and of particular note, about half of adult sexual offenders disclosed that their first sexual offense was committed during their adolescence (Abel, Mittelman, & Becker, 1985; Groth, Longo, & McFadin, 1982). With regard to delinquency, a recent review of the empirical literature (see Seto & Lalumière, 2006) found that adolescent sexual offenders generally had their first official contact with the criminal justice system between 12 and 13 years of age and had substantial criminal histories as well as other conduct problems (although these antisocial histories were less extensive relative to nonsexual offenders). Caldwell (2007) also reported that juvenile sexual offenders were 10 times more likely to engage in nonsexual than sexual recidivism. Although the extant evidence suggests that, for both adults and juveniles, sexual recidivism has a relatively low base rate, criminal versatility (i.e., subsequent nonsexual recidivism) may still be a significant clinical issue (e.g., Hanson & Morton-Bourgon, 2005).

Researchers have hypothesized that some adolescent sexual offenders have deviant sexual interests, whose sexual aggression is part of a wider repertoire of antisocial behaviors (Becker, 1988; Seto & Barbaree, 1997), and this is consistent with the risk factors for sexual recidivism suggested in literature regarding adult sexual offenders (e.g., Hanson & Bussière, 1998, Hanson & Morton-Bourgon, 2005; Quinsey, Lalumière, Rice, & Harris, 1995). In addition, research has shown that adolescent sexual offenders who had committed only sexual offenses were generally better adjusted, had significantly fewer childhood behavioral problems, more prosocial attitudes, and were at lower risks of future delinquency than those who are criminally versatile (Butler & Seto, 2002). In contrast, studies suggest that substantial proportions of adolescent sexual offenders display criminal versatility and therefore have a history of nonsexual offenses (Ryan, Miyoshi, Metzner, Krugman, & Fryer, 1996), commonly associated with a diagnosis of conduct disorder (Bladon, Vizard, French, & Tranah, 2005; France & Hudson, 1993).

#### *Within the Singaporean Context*

In spite of the growing evidence that there are differences between the types of adolescent sexual offenders in western countries, there is currently a dearth of research on adolescent sexual offenders within an Asian context. Singapore is an independent island-state in Southeast Asia, whose population of 4.68 million is composed of 74.8% Chinese, 13.5% Malays, 9% Indians, and 1.7% other ethnic origin (Singapore Department of Statistics, 2007). In 2006, sexual offenses accounted for more than 4% of all crimes in Singapore; these were mostly recorded as molestation offenses (Singapore Police Force, 2007).

#### *Present Study*

Within this context, the aims of this study were (a) to explore the characteristics of the adolescent sexual offenders in Singapore; (b) to explore whether there were any differences between adolescent sexual offenders who had committed only sexual offenses (specialists) as compared with more criminally versatile adolescent sexual offenders, that is, those with other types of offenses (generalists); and (c) to explore the relationship between the recidivism rates and typology (i.e., specialists vs. generalists) within this cohort of adolescent sexual offenders.

We sought to test the following hypotheses: (a) generalists would significantly differ from specialists in terms of background and offending characteristics; and (b) generalists would be significantly more likely to reoffend than specialists.

### Method

#### *Source Sample*

All adolescent males charged with, and convicted of, sexual offenses are ordered by the courts to undergo psychological evaluation at the Clinical and Forensic Psychology Branch (previously known as the Psychological Services Unit) or the Institute of Mental Health in Singapore. Although a small number of the adolescent sexual offenders were referred to the Institute of Mental Health from 1996 to 1998, the vast majority of the adolescent sexual offenders received court-mandated psychological evaluations at the Clinical and Forensic Psychology Branch thereafter. The source sample therefore comprised 156 adolescent males (aged between 12 and 18 years) who were charged with and found guilty of sexual offenses (i.e., molestation, rape, and sodomy), and referred to the Clinical and Forensic Psychology Branch of the Ministry of Community Development, Youth and Sports between June 1996 and January 2007.

*Offender classification.* Adolescent sexual offenders who were charged and convicted with sexual *and* nonsexual offenses (in the past and/or at the point of the psychological evaluation) were classified as "generalists" ($n = 77$)[1]; whereas those adolescent offenders who had committed only sexual offenses, and did not have any previously or concurrently charged and convicted nonsexual offenses at the point of the psychological evaluation (i.e., no involvement in any nonsexual delinquency) were classified as "specialists" ($n = 71$).[1]

### *Ethics*

Ethical approval for the research was obtained through the Clinical and Forensic Psychology Branch of the Ministry of Community Development, Youth and Sports before the commencement of study.

### *Procedure*

Information was collected from multiple data sources that included (a) psychological reports, (b) presentencing reports, (c) charge sheets, (d) statement of facts, and (e) previous assessment and treatment reports. Psychological interviews conducted at the Clinical and Forensic Psychology Branch typically follow a standardized semistructured interview schedule. Hence, the resultant psychological reports usually contained specific information pertaining to several key areas of assessment (e.g., personal and family histories, psychiatric history, as well as sex offending and other criminal offending histories) as well as inclusion of the results from the necessary psychometric instruments.

The major coding categories comprised the following:

1. *Sociodemographic characteristics*: Age at first convicted offense, education level, ethnicity, and family structure (intact family of origin vs. nonintact family of origin [i.e., parents were divorced/separated, or the adolescents were orphaned, adopted or fostered]).
2. *Offender characteristics*: Past nonsexual offense history (convicted offenses), presence of intellectual deficiency, presence of psychiatric condition, self-reported sexual victimization history, and self-reported exposure to pornography.
3. *Offense characteristics*: Age of victims (prepubescent [i.e., 12 years old or younger] vs. pubescent [i.e., older than 12 years]), victim preference (peer-aged [i.e., not more than 2 years difference from the offender's age], non-peer-aged, or mixed), offender–victim relationship (stranger vs. acquaintance [i.e., friends, family members, and relatives]; familial [i.e., family members and relatives] vs. nonfamilial), use of aggression during the sexual offense (i.e., verbally abusing, threatening, physically hitting, and/or restraining the victim), use of weapon during the sexual offense, and the gender of victims (male, female, or both).
4. *Recidivism information*: Recidivism information was obtained from a criminal records check with Singapore Police Force's Criminal Record Office. In parti cular, the specifics of the criminal convictions were detailed in the criminal records check completed on February 1, 2008, and the following categories of recidivism were adopted in this study: sexual recidivism (e.g., indecent exposure, molestation, peeping, rape, and sodomy), violent recidivism (e.g., causing bodily harm, rioting, and robbery), nonviolent offense (e.g., burglary, drug use, fraud, theft, and consensual sex with an underaged girl), and any recidivism (which includes sexual, violent, and other offenses). The average length of the recidivism follow-up was 64.78 months (*SD* = 35.09), but varied between 12 and 139 months.

This study was retrospective in design, so it relied on existing data not necessarily designed or collected for the specific purposes of these particular research questions. Where discrepancies between data sources arose, we relied on information contained in the most recent psychological report (i.e., the report submitted to the courts to assist with disposition). The first author, a psychologist, and two research assistants were involved in the coding of the variables. The psychologist and research assistants each were given an hour of briefing, with each instructed to code specific variables (i.e., they were tasked to code sociodemographic, offender, or offense information). As such, interrater reliability was not formally examined with regard to the coding process. The key terms in this study were operationalized to minimize the likelihood of subjective bias in coding process, and any difficulties encountered during the coding process were referred to the first author for discussion and resolution. All data coders were blind to the recidivistic outcomes, which were sourced from the Criminal Record Office after the initial round of coding. The first author had subsequently coded the recidivism outcome data.

Information relating to the sociodemographic information and offender characteristics was coded from official records. The presence of psychiatric conditions and intellectual deficiency were coded from formal diagnoses that were available in the psychological assessments or case file materials. These psychiatric diagnoses were derived either from the *International Classification of Diseases, 10th edition (ICD-10*; World Health Organization, 1992) or the *Diagnostic and Statistical Manual of Mental Disorders, 4th Edition* (*DSM-IV*; American Psychiatric Association, 1994) classification systems. The adolescent sexual offenders were classified as "intellectually deficient" if they achieved a full scale IQ of less than 70 on the Wechsler Intelligence Scale for Children–Third Edition (WISC-III; Wechsler, 1991). However, it should be noted that the WISC-III was only administered where clinically indicated. The presence of self-reported history of sexual victimization and self-reported use of pornography (which included viewing pornographic images from magazines, video clips, or Internet Web sites) was coded from psychological reports.

### Statistical Analyses

The sample was characterized using descriptive statistics, with categorical data reported as numbers and percentages, and continuous data presented in relation to the mean and standard deviation. Histograms of the continuous data were plotted to check for skewed distributions. Univariate analyses sought to compare the characteristics of the specialists and generalists, as well as the recidivists and nonrecidivists. Chi-square tests of association were computed for categorical data, and two-tailed independent *t* tests were computed for continuous data. Significant univariate associations were modeled using forward stepwise logistic regression to develop a classification model for the sexual offenders. A forward stepwise logistic regression model was also conducted to develop the most parsimonious model for predicting recidivists, based on univariately significant variables (i.e., removing those variables that become nonsignificant once the effects of other variables have been controlled for in the models). Transformation was conducted on the covariates (continuous variables) of the regression models if they exhibited severe skewness and kurtosis. The area under curve (AUC) of the receiver operating characteristics (ROC) curve was plotted as an indication of the classification accuracy of the resultant model (Mossman, 1994), and the "goodness-of-fit" test of the models checked using the Hosmer–Lemeshow test (Agresti, 1996). Additionally, to consider factors associated with time to recidivism, Cox regression models were computed to compare the recidivistic outcomes of the generalists and specialists. The end points were a recidivistic act[2] or the end of the follow-up period, with results reported as hazard ratios. Analyses were carried out using the SPSS Version 16.

## Results

### Sample Characteristics

The average age of the overall sample when referred for assessment was 15 years (*SD* = 1.37, range = 12-18 years). The majority had received at least secondary-level mainstream education (124/144,[3] 86.1%) and came from an intact family of origin (99/153,[3] 64.7%). The overall sample also largely comprised Chinese (69/156, 44.2%) and Malays (58/156, 37.2%).

Table 1 summarizes the offender characteristics of the overall sample and the specifics of the offenses. The average age of the adolescent sexual offenders at their first convicted sexual offense was 14.28 years (*SD* = 1.40, range = 11-18 years), and 30.8% (44/143) had a past offense history. Adolescents who assaulted prepubescent victims were more likely to target their family members and relatives, $\chi^2$ (1, *N* = 138) = 16.46, $p < .001$, and thus less likely to assault strangers, $\chi^2$ (1, *N* = 150) = 5.40, $p = .02$. The average age of victims was 15.88 years (*SD* = 6.79, range = 3-46) and was normally distributed. The majority of the sample (104/148,[2] 70.3%) used aggression during their sexual offenses, but only 3 (2%) reportedly used weapons.

More than a third (55/156) of the sample reoffended during the course of the follow-up period; 18 (11.5%) were convicted of sexual offenses, 18 (11.5%) violently offended, 32 (20.5%)[4] were convicted of sexual and/or violent offenses,[5] and 44 (28.2%) were convicted of other nonviolent offenses (e.g., theft-related, drug offenses, or consensual sex with underaged girls; see Table 2). Notably, nine sexually reoffended during their initial court orders and two were reconvicted on multiple occasions during follow-up. In addition, 5 of the violent recidivists and 18 of the nonviolent recidivists reoffended during their court orders.

**Table 1.** Offender and Offense Characteristics for the Overall Sample, Specialists, and Generalists

| | Overall ($N^a$ = 156) | | Specialists ($n$ = 71) | | Generalists ($n$ = 77) | | |
|---|---|---|---|---|---|---|---|
| Variables | Mean | *SD* | Mean | *SD* | Mean | *SD* | *p* |
| Follow-up period (months) | 64.78 | 35.09 | 56.99 | 31.33 | 67.83 | 36.55 | |
| Time-at-risk period (months) | 31.73 | 30.29 | 28.12 | 27.61 | 30.77 | 29.86 | |
| Age when referred (years) | 15.00 | 1.37 | 15.01 | 1.43 | 14.89 | 1.30 | |
| Age at first convicted sexual offense (years) | 14.28 | 1.40 | 14.38 | 1.35 | 14.09 | 1.41 | |
| Number of sexual offenses | 2.76 | 2.79 | 2.93 | 3.24 | 2.70 | 2.41 | |
| Total number of offenses | 4.23 | 3.87 | 2.78 | 2.67 | 5.53 | 4.31 | b |
| | $N^a$ | Percentage | $n^a$ | Percentage | $n^a$ | Percentage | |
| Exposed to pornography | 98/137 | 71.5 | 45/65 | 69.2 | 49/67 | 73.1 | |
| Reportedly sexually victimized | 9/156 | 5.8 | 6/71 | 8.5 | 3/77 | 3.9 | |
| Has psychiatric condition | 5/143 | 3.3 | 2/67 | 2.9 | 3/71 | 4.1 | |
| Intellectually deficient | 19/151 | 12.6 | 9/69 | 13 | 9/74 | 12.2 | |
| Assaulted multiple victims | 64/148 | 43.2 | 30/69 | 43.5 | 32/73 | 43.8 | |
| Assaulted stranger victims | 80/150 | 51.3 | 33/69 | 47.8 | 43/74 | 58.1 | |
| Assaulted familial victims | 12/150 | 8 | 10/69 | 14.5 | 1/73 | 1.4 | c |
| Assaulted prepubescent victims | 57/153 | 37.3 | 29/70 | 41.4 | 26/76 | 34.2 | |
| Ever assaulted male victims | 11/153 | 7.2 | 3/70 | 4.3 | 8/76 | 10.5 | |
| Use of physical and verbal aggression during sexual offense | 104/148 | 70.3 | 42/69 | 60.9 | 57/73 | 78.1 | |

Note: Assault refers to sexual assault.

a. Because of missing data there were differences in the denominators, and eight sexual offenders were not also classified.

b. Although this difference is significant at $p < .001$, it is likely an artifact of the typological classification and thus left out of comparisons.

c. Difference between specialists and generalists was significant after making Bonferroni adjustment ($p < .0042$).

**Table 2.** Univariate Comparison of Recidivistic Outcomes Across Typology

| | Overall ($N^a$ = 156) | | Specialists ($n$ = 71) | | Generalists ($n$ = 77) | | |
|---|---|---|---|---|---|---|---|
| Recidivistic Outcomes | $N^a$ | Percentage | $n^a$ | Percentage | $n^a$ | Percentage | $p$ |
| Sexual recidivism | 18/156 | 11.5 | 7/71 | 9.9 | 11/77 | 14.3 | |
| Violent recidivism | 18/156 | 11.5 | 1/71 | 1.4 | 14/77 | 18.2 | ** |
| Sexual and/or violent recidivism | 32/156 | 20.5 | 8/71 | 11.3 | 21/77 | 27.3 | * |
| Nonviolent recidivism | 44/156 | 28.2 | 12/71 | 16.9 | 29/77 | 37.7 | ** |
| Any recidivism | 55/156 | 35.3 | 17/71 | 23.9 | 35/77 | 45.5 | ** |

Note: Recidivism statistics included convicted recidivistic acts that were committed during probation and/or residential orders.
a. As a result of missing data, there were differences in the denominators, and eight sexual offenders were not also classified.
*Difference between specialists and generalists was significant at $p < .05$ level.
**Difference between specialists and generalists was significant at $p < .01$ level.

#### *Comparing the Characteristics of Specialists and Generalists*

Table 1 summarizes the comparison of the characteristics of specialists and generalists. Initial univariate analyses (with Bonferroni adjustment) indicated that sexually assaulting familial victims was significantly associated with typology, $\chi^2$ (1, $N$ = 143) = 8.68, exact $p$ = .004. This variable remained significant even when applied to a forward logistic regression model with age at first sexual offense and total number of offenses as covariates. Specifically, the model suggested that generalists were much less likely than the specialists to sexually assault familial victims (OR [odds ratio] = 0.01; 95% CI [confidence interval] = <0.01-0.40). This simple model correctly classified 74% of the sample, and of note, the model was better at correctly classifying generalists (76% correct) as opposed to specialists (71% correct). The Hosmer–Lemeshow test suggested no evidence of a lack of fit with this model, $\chi^2$(7, $N$ = 137) = 11.35, $p$ = .124.

With regard to recidivism follow-up, univariate analyses showed that specialists and generalists did not differ significantly with respect to rates of sexual recidivism, but the generalists were more likely to engage in violent, sexual and/or violent,[5] nonsexual, and nonviolent, as well as any criminal recidivism (see Table 2). Further analyses using Cox regression models, controlling for the potentially confounding effect of total number of convicted offenses and age at first convicted sexual offense, revealed that generalists were 9.31 times (95% CI = 1.14-76.39) more likely than specialists to engage in violent recidivism following the completion of their court orders (see Table 3 and Figure 1).

### Discussion

Adolescents committed about 5% of the sexual offenses in Singapore in 2006 (Ministry of Community Development, Youth and Sports, 2006). Juvenile sexual offending therefore

**Table 3.** The Likelihood of Generalists Having Engaged in Different Forms of Recidivism as Compared With Specialists

| Recidivistic Outcomes | Hazard Ratio | 95% Confidence Interval | *p* |
|---|---|---|---|
| Sexual recidivism | 0.63 | 0.15-2.57 | |
| Violent recidivism | 9.31 | 1.14-76.39 | * |
| Sexual and/or violent recidivism | 1.96 | 0.70-5.50 | |
| Nonviolent recidivism | 2.27 | 0.86-6.01 | |
| Any recidivism | 2.10 | 0.93-4.71 | |

Note: Total offenses and age at first convicted sexual offense have been adjusted for in these Cox regression models.
*Difference between specialists and generalists was significant at $p < .05$ level.

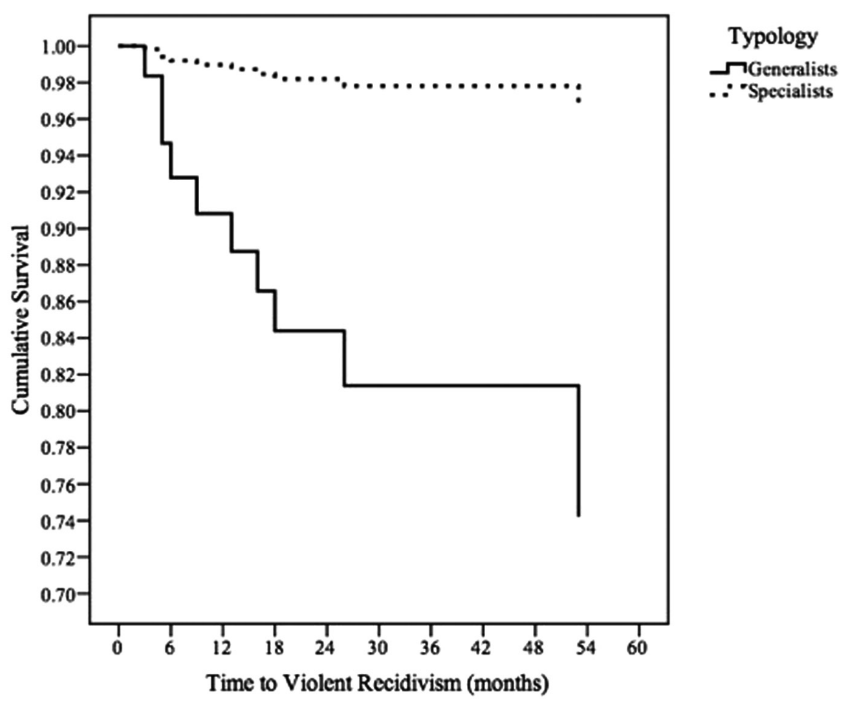

**Figure 1.** Survival curves for generalists and specialists (violent recidivism)

clearly represents an ongoing social, judicial, clinical, and policy issue for services in Singapore, as well as elsewhere. This study describes the characteristics of a sizeable cohort of adolescents charged with and convicted of sexual offenses over an 11.5-year period. An examination of the typology within this group of adolescent sexual offenders (i.e., specialists vs. generalists) produced several noteworthy differences and raises some clinical concerns about the identification and management of those who display criminally versatile offending patterns.

### Limitations

Before discussing the results of this study any further, it is important to note several limitations with this study that may have an impact on the generalizability of the findings. First, this is a retrospective study and had relied on data collected from a number of data sources, which, by definition, were not necessarily primarily intended for research application. Therefore, there were missing data on some of the variables for some of the cases, and the quality of the data available in the official records was variable at times. Second, reliance on the electronic data for recidivism follow-up will inevitably lead to an underestimate of offending because of the further offenses not having been identified, charged, and convicted.

Third, although the sample consisted of all referrals to the Clinical and Forensic Psychology Branch between June 1996 and January 2007, we cannot be entirely confident that this sample included every adolescent charged and convicted of such offenses during this time period. From 1999 onward, the courts referred most of the adolescents who were charged and convicted of sexual offenses to the service, so we can be relatively confident of the completeness of this group. However, the service was not the exclusive referral source prior to this time. As such, a small number of individuals may have been referred to the local state mental hospital for assessment and/or treatment instead of the Clinical and Forensic Psychology Branch. Last, specific information on youth sexual offending rates is unavailable to the authors at this point in time for comparisons with the trends from Western countries. Notwithstanding the aforementioned limitations, this study describes the characteristics of what equates to a fairly large group of adolescents convicted of sexual offenses (i.e., molestation, rape, and sodomy). Collectively, these findings have important implications for clinical practice.

### Comparisons With Previous Studies

The data presented in this study generally replicate some previously published findings, which appear to indicate that these findings have some robustness across cultures. The characteristics of this group of adolescent sexual offenders in Singapore were, broadly speaking, similar to those reported in previous studies in other western countries with respect to age (e.g., Hunter, Figueredo, Malamuth, & Becker, 2004; Waite et al., 2005) and criminal histories of the offenders (Nisbet et al., 2004), victim characteristics (Hummel, Thömke, Oldenbürger, & Specht, 2000), and recidivism data (e.g., Caldwell, 2007; Worling & Curwen, 2000). Similar to previous studies (Butler & Seto, 2002; Hunter et al., 2004, Worling, 1995), our current findings also indicate that those adolescent offenders who had sexually assaulted prepubescent victims tended to target their family members or relatives.

However, some interesting differences were found when considering the extant western literature. For example, although adolescent sexual offenders commit 5% of the sexual offenses in Singapore, their counterparts in North America are responsible for about 20% of the sexual offenses (Federal Bureau of Investigation, 2002). Another striking difference is that majority of the research from Western countries showed that adolescent sexual offenders were generally less likely to sexually assault strangers (e.g., Davis & Leitenberg, 1987; Fehrenbach, Smith, Monastersky, & Deisher, 1986; Vizard, Hickey, French, & McCrory, 2007), whereas more than half of this Singaporean sample sexually offended against strangers. In addition, the present sample of adolescent sexual offenders was much less likely to have offended against male victims as compared with published Western findings (Kemper & Kistner, 2007; Nisbet et al., 2004; Vizard et al., 2007).

There were also much lower self-reported rates (71.5%) of exposure to pornography in this adolescent sample, as compared with a North American study of adolescent sexual offenders, whereby about 90% of the adolescent sexual offenders had reported being exposed to pornography (Zgourides, Monto, & Harris, 1997). Moreover, some studies have suggested an association between previous physical and sexual victimization experiences and later sexual offending behaviors (e.g., Becker & Hunter, 1997, Ford & Linney, 1995, Hummel et al., 2000). Our data did not support this purported association, with only nine of the adolescent boys indicating a history of sexual victimization. This comparatively low rate of sexual victimization could be because of a low level of disclosure among the adolescents, individual differences in interview style, comfort levels, and rapport between interviewers and the possibility that sexual victimization histories were not routinely asked about in spite of the semistructured interview schedule.

### A Typology of Adolescent Sexual Offenders

There was support for the first hypothesis that generalists and specialists are a valid typological distinction in spite of their similarities. Specifically, generalists were less likely than specialists to sexual offend against their family members and relatives. The model successfully classified almost three quarters of the sample as being a generalist or specialist. In fact, the model was better at identifying generalists, who are considered to have a higher risk trajectory, with more than three quarters being classified as such by the statistical model presented here. Nevertheless, it should be noted that the percentage of adolescent sexual offenders who were correctly classified as generalists or specialists was less than optimal clinically. This may, in part, have been affected by the limited nature of the archival data available here.

In spite of the limitations of the classification model, generalists and specialists were clearly different in terms of their risk trajectories for violent offending, which could be largely attributed to their delinquency status rather than sexual deviance when considering the findings of this study. In particular, the generalists were as likely as the specialists to sexually reoffend, but also significantly more likely to engage in violent recidivism. For example, almost one in five of the generalists had engaged in violent recidivism within 2.5 years of being unsupervised in the community as compared with 2% of the specialists. Generalists were also shown, at a univariate level, to be more likely than specialists to recidivate on a composite index of sexual and/or violent recidivism, but such a difference became nonsignificant after accounting for the age at first convicted sexual offense and the total number of offenses. Furthermore, although Seto and Lalumière (2005) suggested the age of the victims (i.e., child vs. peer-aged or older victims) could provide an explanation for the specialist–generalist typology, we did not find such associations in our study. Taken together, there was partial support for the second hypothesis (i.e., generalists were significantly more likely to reoffend than specialists), thus raising significant issues pertaining to the identification of sexual offenders who also commit violent offenses.

From a clinical perspective, these generalists can be assessed specifically for their risk of sexual offending by means of focusing on sexually deviant interests, previous conduct disorder, and antisocial personality factors if identified early enough in their offending careers (e.g., Butler & Seto, 2002). This may be particularly important in determining the risks that this group poses in relation to committing sexual offenses in adulthood (Nisbet et al., 2004). There is also a clear clinical need to accurately assess the risk of violent or even general offending within this group, which may routinely be neglected because of the focus on the sexual offenses. Such assessments of risk and criminogenic need are important for making informed decisions about the level of supervision and the intensity of treatment required for effective offender rehabilitation (Andrews, Bonta, & Hoge, 1990).

### *Conclusion*

Overall, this study found that more than one third of adolescents, who were convicted of sexual offenses, reoffended during a follow-up of up to 11.5 years, and that violent recidivism was a significant problem within this offender population, especially among the criminally versatile adolescent sexual offenders. Further research should investigate the risk factors that are associated with long-term justice and mental health outcomes of offenders who had (a) participated in sexual offender treatment programs and (b) reoffended during their initial court orders, as well as those dynamic and offense-specific factors (e.g., cognitive distortions, grooming strategies, modus operandi, and types of deviant sexual fantasies) that are associated with sexual offending behaviors using repeated-measures designs. It would also have been useful to examine the developmental trajectory in this group of adolescent sexual offenders over the longer term (Moffitt & Caspi, 2001). Furthermore, it would be interesting to investigate the characteristics and situational factors of the group of recidivists who had reoffended during supervision and/or rehabilitation.

#### Authors' Note

The views expressed are those of the authors and do not represent the official position or policies of the Ministry of Community Development, Youth and Sports or Monash University.

### Acknowledgments

The authors thank the staff of Clinical and Forensic Psychology Branch, Policy Development Branch, and Strategic Policy and Research Division of the Ministry of Community Development, Youth and Sports for their support; in particular, Jennifer Teoh for her facilitation of the administrative and logistical arrangements. We would like to acknowledge the contribution of I Ting Tsao for her assistance in the retrieval of archival and follow-up data. We also thank Jia Ying Lim for her comments on this article.

### Declaration of Conflicting Interests

The author(s) declared no conflicts of interest with respect to the authorship and/or publication of this article.

### Funding

The author(s) received no financial support for the research and/or authorship of this article.

## Notes

1. 1. As a result of missing data, eight adolescent sexual offenders were not classified.
2. 2. These recidivistic acts were defined as any convicted sexual, nonviolent, as well as violent offenses in accordance with their recidivistic outcome categories.
3. 3. The differences in the denominators are because of missing data.
4. 4. Four adolescent sexual offenders had reoffended violently and sexually; the rest committed either violent or sexual offenses.
5. 5. This is a composite index of recidivism where the adolescent sexual offender is considered to have recidivated if he committed either violent or sexual offense or both.
